# Epidemiology, clinical outcomes, and factors associated with mortality among cerebrospinal meningitis cases in the Upper West Region of Ghana: an eight-year surveillance study

**DOI:** 10.1186/s12879-026-14070-3

**Published:** 2026-07-22

**Authors:** Abdul-Wahab Chiemina Bakariwie, Andrew Mpagwuni Ziblim, Abdul-Hanan Saani Inusah, Mohammed Rashad Mutawakil, Ireneus Malinbong Kyebambo

**Affiliations:** 1 Department of Internal Medicine, Tamale Teaching Hospital, Tamale, Ghana; 2 Department of Anaesthesia and Intensive Care, University of Ghana Medical Centre, Legon, Ghana; 3 Department of Internal Medicine, Upper East Regional Hospital, Bolgatanga, Ghana; 4 Department of Health Promotion, Education and Behavior, Arnold School of Public Health, University of South Carolina, Columbia, SC USA; 5 Planning Unit, Local Government Service, Zabzugu District Assembly, Zabzugu, Ghana; 6 Department of General Surgery, Wa Municipal Hospital, Wa, Ghana

**Keywords:** Cerebrospinal meningitis, Bacterial meningitis, Disease surveillance, Mortality, Clinical presentation, Ghana, Meningitis belt, Upper West Region, Epidemiology, Delay to consultation

## Abstract

**Introduction:**

Cerebrospinal meningitis is a clinical syndrome with multiple etiologies, however, in sub-Saharan Africa, infectious and contagious forms remain highly prevalent and carry high risks of mortality and disability. The Upper West Region of Ghana is found within the meningitis belt and frequently records the highest numbers of incident meningitis cases within the country yearly.

**Methods:**

This study analyzed all routinely-collected data on surveillance-defined suspected and confirmed cases of meningitis in the Upper West Region of Ghana between January 2016 and December 2023. The data were obtained from the Disease Surveillance Unit of the region. Descriptive statistics were used to summarize patient characteristics, clinical presentation, consultation delays, and outcomes. Bivariate (unadjusted) analyses were conducted to explore associations between individual covariates and each outcome of interest, and then multivariable logistic regression models and reported adjusted odds ratios were used to find associations.

**Results:**

407 meningitis–related clinical consultations were recorded. The median age was 13 years (IQR: 4–23 years). More than half of cases (55.3%) occurred among children aged 0–15 years. Males accounted for 55.8% of cases. 32 deaths were recorded during the study period, corresponding to a crude mortality rate of 7.9%. Fever (41.0%), neck stiffness (35.3%), and headache (31.7%) were the most frequently reported symptoms. The median time to first consultation was 2 days (IQR: 1–3 days), and almost half of patients (46.9%) sought care within one day of symptom onset. Those aged ≥ 46 years had significantly higher odds of death (aOR = 6.01, 95% CI: 1.61–22.46). Patients presenting with classic meningitis symptoms had lower odds of death (aOR = 0.38, 95% CI: 0.15–0.97), with patients presenting with other non-specific symptoms having significantly higher odds of death (aOR = 3.84, 95% CI: 1.33–11.05).

**Conclusion:**

Cerebrospinal meningitis affected individuals across all age groups, although the young were disproportionately affected. Older individuals and those with symptoms other than the classic meningitis symptoms had higher odds of mortality. It is important for healthcare workers to recognize this, so that they can maintain a higher index of suspicion especially during outbreaks. Policymakers need to be cognizant of the fact that meningitis remains an important public health concern and that vaccinations; health education and promotion; and targeted interventions for older populations should be prioritized. The public should be educated on the non-specific features of the disease.

**Clinical trial number:**

Not applicable.

**Supplementary Information:**

The online version contains supplementary material available at https://doi.org/10.1186/s12879-026-14070-3.

## Introduction

Cerebrospinal meningitis (CSM) is inflammation of the meninges (coverings of the brain and spinal cord – pia, arachnoid, and dura mater) due to infection from bacteria or less commonly viruses, parasites, and fungi. It can also result from cancer, autoimmune conditions, or drug reactions [1]. CSM can quickly become life-threatening and invariably fatal if not suspected quickly and treated early [2]. The clinical features of CSM have changed very little in the last 100 years. The so-called classic symptoms of fever, neck stiffness, and headaches were observed and documented with the first reported case and continue to be characteristic [3], although there are various manifestations of the disease such as altered consciousness, seizures, general body pain, and vomiting.

CSM, like many other infectious diseases in the present day, has seen a decrease in absolute numbers and disability-adjusted life-years (DALYs) [4]. Globally, even though incident cases increased from 2.5 million in 1990 to 2.82 million in 2016, deaths decreased by 21% in that same period, mostly as a consequence of vaccinations conferring herd immunity, and effective antibiotic therapy [3]. Worldwide, the highest mortality rates are found within the meningitis belt [1], where incidence rates can reach 1000 cases per 100 000 people [5], in contrast to the global incidence of 39 in 100 000 [4]. The concept of a meningitis belt was first proposed and described in 1963. This region spans the Sahel and sub-Sahel, extending from Senegal in West Africa to Ethiopia in East Africa [6]. More recently, an increasing number of outbreaks have been reported outside this area, suggesting a possible expansion of the belt, potentially linked to climate change. The peculiar features of CSM outbreaks, namely: seasonality, geographical limitations, and periodicity, have persisted from the first documented cases over a century ago to this day [3]. Despite global declines in mortality, mortality from CSM remains disproportionately high in the meningitis belt [7–9].

The Gold Coast (now Ghana) recorded its first outbreak between 1906 and 1908 [10]. In Ghana, meningitis outbreaks are frequently reported in the northern parts of the country which fall under the meningitis belt. Dry and hot weather conditions account for the disparity in incidence of the disease between the north and south of Ghana [7]. Indeed, incidence of meningitis in Ghana is highest in the three northernmost regions of the country, with the Upper West Region (UWR) recording prevalence of 18.0%–38.8% [7] during outbreaks. This is attributed to the low humidity and high winds in that area which contribute to breakdown of mucosal defenses against the common pathogens that cause the disease [11]. Although the case fatality rates in Ghana have been dropping steadily from 20% to 47% between 1998 and 2015 to 0.8%‒28.6% between 2016 and 2021 [7, 8, 12], the fact remains that CSM can be a deadly condition, and treatment delay is associated with increased mortality [13].

In this study, in order to understand the peculiar dynamics of CSM in a high-burden area such as the UWR of Ghana, we assessed the epidemiology of CSM in the region from January 2016 to December 2023 as well as the associations between mortality and a) patient characteristics, b) delay to presentation, and c) symptoms.

## Methods

### Data source and study setting

This study used routinely collected surveillance data on suspected and confirmed cases of CSM reported in the UWR of Ghana between January 2016 and December 2023. Although surveillance records were available from 2015, only six cases were recorded that year, with substantial missing data. Therefore, analyses were restricted to the 2016–2023 period to ensure data completeness and analytic consistency.

The UWR lies within the African meningitis belt and experiences recurrent seasonal outbreaks of CSM. The population of the UWR is 901,502, with 74% of this number living in rural areas [14]. The region is bordered by Burkina Faso to the north, Ivory Coast to the west, the Savannah Region to the south (carved out of the Northern Region in 2019) and the Upper East Region to the east.

The data were obtained from the Upper West Regional Disease Surveillance Unit, which maintains surveillance records compiled through Ghana’s Integrated Disease Surveillance and Response (IDSR) system and the District Health Information Management System (DHIMS). These systems capture standardized line-list information on epidemic-prone diseases reported from health facilities across the region. The dataset included records from public health facilities across ten districts in the region. No sampling was conducted; the analytic dataset represents all reported CSM-related clinical consultations recorded in the surveillance system during the study period.

The analytic dataset included both suspected and laboratory-confirmed CSM cases reported through the regional surveillance system. Suspected cases were retained in accordance with surveillance case definitions used in routine reporting. Cases were included based on reporting health facilities within the Upper West Region rather than patients’ administrative district of residence. As a result, the dataset reflects cases managed within the Upper West regional healthcare system, including referrals from two districts in the Savannah Region (Bole Bamboi and Sawla-Tuna-Kalba). No cases from Sissala East and Sissala West (both districts within the Upper West Region) were captured in this dataset during the study period.

### Case definition

Case classification followed standard meningitis surveillance practices used in Ghana’s IDSR system.

A *suspected case* was defined as any individual presenting with acute onset of illness and at least one clinical feature suggestive of meningeal involvement, including neck stiffness, altered consciousness, convulsions, or other features of meningeal irritation, as recorded at first healthcare contact. Additionally, given the outbreak context, individuals identified through active contact tracing were given medical consultation. This means that those evaluated during the ultra-early or prodromal phase of infection may have had no documented symptoms at the time of initial assessment, but were retained in the surveillance dataset due to known high-risk exposure and subsequent clinical progression.

A *confirmed case* was defined as a suspected case with laboratory evidence of meningitis based on cerebrospinal fluid (CSF) analysis, including organism identification through Gram stain or culture.

Not all suspected cases underwent lumbar puncture due to clinical or logistical constraints. Therefore, both suspected and confirmed cases were retained to reflect the surveillance-defined burden of disease in the region.

### Study variables

#### Outcomes

The primary outcome was mortality, defined as death during the clinical episode (coded as died vs. survived). The secondary outcome was delay to consultation, defined as the number of days between self-reported symptom onset and first presentation to a health facility. Delay-to-consultation data were unavailable for some observations because dates of symptom onset, first consultation, or both dates were missing from the surveillance records. Delay to consultation was summarized descriptively as both a continuous variable (days) and a categorical variable (0–1 day, 2–3 days, 4–7 days, and > 7 days). In regression analyses, delay was modeled as a continuous variable.

#### Independent variables

Independent variables were selected based on clinical relevance and availability in the surveillance dataset. These included demographic characteristics (age and sex), clinical presentation at first consultation, and laboratory confirmation status. Age was categorized into three groups: 0–15 years, 16–45 years, and ≥ 46 years. Sex was recorded as male or female.

Clinical symptoms recorded at first presentation included fever, headache, neck stiffness, altered consciousness, convulsions, and other reported symptoms. For analytic purposes, symptoms were grouped based on the established clinical presentation of meningitis and common practice in outbreak surveillance. Specifically, *classic meningitis symptoms* included fever, headache, and neck stiffness; *neurological manifestations* included altered consciousness and/or convulsions; and other non-specific symptoms comprised all remaining reported symptoms, including vomiting, body weakness, body pains, cough, and other less specific clinical complaints.

Symptoms were recorded as binary outcomes (“Yes” or “No”) for each of the symptom categories in the surveillance dataset, including an “Others” category for non-specified complaints. Consequently, records with a “No” across all categories were analytically classified as having **no** symptoms recorded in the available surveillance symptom fields. In the Ghana Health Service context, surveillance records arise through routine clinical presentation, active outbreak screening, or contact tracing. Because the dataset lacked a specific variable for the route of entry, we could not systematically separate these paths. However, nearly half (46.2%, *n* = 60) of all laboratory confirmed cases in our cohort presented with no documented symptoms at their first consultation. This epidemiological pattern indicates that active contact tracing successfully captured and fast-tracked high-risk individuals for medical evaluation during the ultra-early prodromal phases of infection, before overt clinical signs manifested. While minor clinical misclassification or missed recording cannot be entirely ruled out, these cases were conservatively retained to preserve the true surveillance-defined burden and clinical trajectory of the epidemic.

In addition, an exploratory symptom-count variable was constructed based on the number of symptoms recorded in the surveillance dataset. Categories were defined as no symptoms recorded = 0, low symptom count = 1 symptom, moderate symptom count = 2–3 symptoms, and high symptom count = 4 or more symptoms. All recorded symptoms were weighted equally because the surveillance dataset did not include standardized information on symptom severity, duration, timing, or clinician-rated severity. Therefore, this variable reflects the number and breadth of recorded symptoms, not clinical severity.

Laboratory confirmation status was classified as confirmed CSM or suspected/unconfirmed CSM, based on available laboratory results recorded in the surveillance system. Laboratory confirmation results were unavailable for some consultations because laboratory records were incomplete or missing in the surveillance dataset.

### Statistical analysis

Descriptive statistics were used to summarize patient characteristics, clinical presentation, consultation delays, and outcomes. Continuous variables were summarized using medians and interquartile ranges, while categorical variables were summarized using frequencies and percentages.

Bivariate (unadjusted) analyses were first conducted to explore associations between individual covariates and each outcome of interest. These analyses were used for descriptive screening and to inform subsequent multivariable model specifications. Results from bivariate analyses are presented in Supplementary Table S1.

To examine factors independently associated with mortality among patients with suspected or confirmed cerebrospinal meningitis, we estimated multivariable logistic regression models and reported adjusted odds ratios (aORs) with corresponding 95% confidence intervals (CIs). The multivariable model presented in Table 2 represents the primary inferential analysis for mortality and included both suspected and laboratory-confirmed cases recorded in the surveillance system. Covariates included in this model were selected a priori based on data availability in the surveillance system, clinical relevance, and evidence of association in bivariate analyses.

Because the surveillance data included relatively small numbers of deaths in some strata and potential clustering by district, we conducted sensitivity analyses to assess the robustness of the mortality model. These included Firth’s penalized logistic regression to address potential small-sample bias and quasi-separation, as well as logistic regression models with district fixed effects and robust standard errors to account for unobserved district-level heterogeneity. Results from the robustness checks are presented in Supplementary Table S2.

As a secondary analysis, we examined factors associated with delay to consultation. Delay was modeled as a continuous outcome. The primary model for this analysis was ordinary least squares (OLS) linear regression with robust standard errors, presented in Table 3. This approach was selected to allow direct interpretation of regression coefficients as differences in days to presentation.

To assess the stability of findings for delay to consultation, we conducted additional sensitivity analyses, including OLS regression with bootstrap standard errors and an alternative generalized linear model (GLM) specification. Bootstrapped OLS was used to relax distributional assumptions and obtain robust standard errors through resampling, while the GLM was fitted as a robustness check because it does not require normality of the dependent variable and allows alternative variance specifications. These models were used to evaluate whether conclusions were sensitive to distributional assumptions or variance estimation methods. Results from these analyses are presented in Supplementary Table S3.

Missing data were handled using listwise deletion in multivariable models. Due to this approach, the final estimation sample sizes differed slightly across models. To assess potential bias related to missing data, we compared included and excluded observations, and reported in Supplementary Table S4.

Model diagnostics were assessed using post-estimation measures, including the Akaike Information Criterion (AIC), and multicollinearity was evaluated using variance inflation factors (VIFs). All VIF values indicated low risk of multicollinearity.

All analyses were conducted using Stata version 14.1, and statistical significance was assessed at the 5% level.

## Results

### Descriptive characteristics

A total of 407 CSM–related clinical consultations were recorded in the Upper West Region between 2016 and 2023 (Table 1). The median age was 13 years (IQR: 4–23 years), reflecting a right-skewed age distribution with a substantial burden among pediatric populations. More than half of cases occurred among children aged 0–15 years (55.3%), followed by those aged 16–45 years (33.9%) and ≥ 46 years (10.8%). Males accounted for 55.8% of cases. Overall, 32 deaths were recorded during the study period, corresponding to a crude mortality of 7.9%. Among the 32 deaths recorded, the largest proportion occurred among individuals aged 16–45 years, accounting for 56.3% of all deaths. However, age-specific mortality differed markedly across groups. Patients aged ≥ 46 years experienced the highest mortality risk, with 15.9% of cases resulting in death, followed by those aged 16–45 years (13.1%). In contrast, children aged 0–15 years had substantially lower mortality (3.1%), despite representing over half of all reported cases.

**Table 1:** Descriptive characteristics of suspected and confirmed cerebrospinal meningitis cases in the Upper West Region of Ghana, 2016–2023 (*N* = 407)

Variable	Number (%)
Age (years)	
Median (IQR)	13 (4–23)
**Age Group**	
0–15	225 (55.3)
16–45	138 (33.9)
> 46	44 (10.8)
**Sex**	
Male	227 (55.8)
Female	180 (44.2)
**Mortality**	
Died	32 (7.9)
Survived	374 (92.1)
**Laboratory result**	
Confirmed CSM	130 (37.7)
Suspected CSM	215 (62.3)
Clinical symptoms*	
Fever	149 (41.1)
Headache	115 (31.7)
Neck stiffness	128 (35.3)
Altered consciousness	20 (5.5)
Convulsion	52 (14.3)
Vomiting	36 (9.9)
Body weakness	9 (2.5)
Body Pains	28 (7.7)
Cough	8 (2.2)
Others	51 (12.1)
**Clinical presentation (grouped)**	
Classical symptoms	216 (53.1)
Neurological symptoms	70 (17.2)
Other symptoms	106 (26.0)
Symptom Count*	
No symptoms recorded	121 (29.7)
Low symptom count (1)	10 (2.5)
Moderate symptom count (2–3)	72 (17.7)
High symptom count (4+)	204 (50.1)
**Delay to consultation (days)**	
Median (IQR)	2 (1–3)
**Delay categories**	
0–1 day	183 (46.9)
2–3 day	138 (35.4)
4–7 day	54 (13.9)
> 7 day	15 (3.9)
**District of consultation**	
Bole Bamboi District**	1 (0.3)
Daffiama/Bussie/Issa District	6 (1.3)
Jirapa Municipal	2 (0.5)
Lawra Municipal	1 (0.3)
Nandom Municipal	1 (0.3)
Nadowli Kaleo District	22 (5.4)
Sawla-Tuna-Kalba District**	11 (2.7)
Wa East District	57 (14.0)
Wa Municipal	178 (43.7)
Wa West District	128 (31.5)

Values are presented as number (%) unless otherwise stated. Clinical symptoms are not mutually exclusive. Classical meningitis symptoms include fever, headache, and neck stiffness

Neurological manifestations include altered consciousness and/or convulsions

Symptoms reflect those documented at first healthcare presentation

* Symptom count reflects the number of symptoms recorded in the surveillance dataset and should not be interpreted as a clinical severity score

** Districts are located in the neighboring Savannah Region

Totals may not equal 407 for some variables because of missing data. Mortality outcome data were available for 406 consultations, laboratory confirmation results for 345 consultations, delay-to-consultation data for 390 consultations, and grouped clinical presentation data for 392 consultations

Approximately one-third of cases (37.7%) were laboratory-confirmed CSM, while the remainder were classified as suspected cases. Fever (41.0%), neck stiffness (35.3%), and headache (31.7%) were the most frequently reported symptoms. Altered consciousness and convulsions were reported in 5.5% and 14.3% of cases, respectively.

When symptoms were grouped by clinical presentation, 53.1% of patients presented with classic meningitis symptoms, 17.2% with neurological manifestations, and 26.0% with other non-specific symptoms. Based on the exploratory symptom-count variable, 50.1% of patients had a high symptom count (4 or more symptoms recorded), while 29.7% had no symptoms recorded in the available surveillance symptom fields.

The median time to first consultation was 2 days (IQR: 1–3 days). Almost half of patients (46.9%) sought care within one day of symptom onset, whereas 35.4% presented after 2–3 days.

**Fig. 1:**
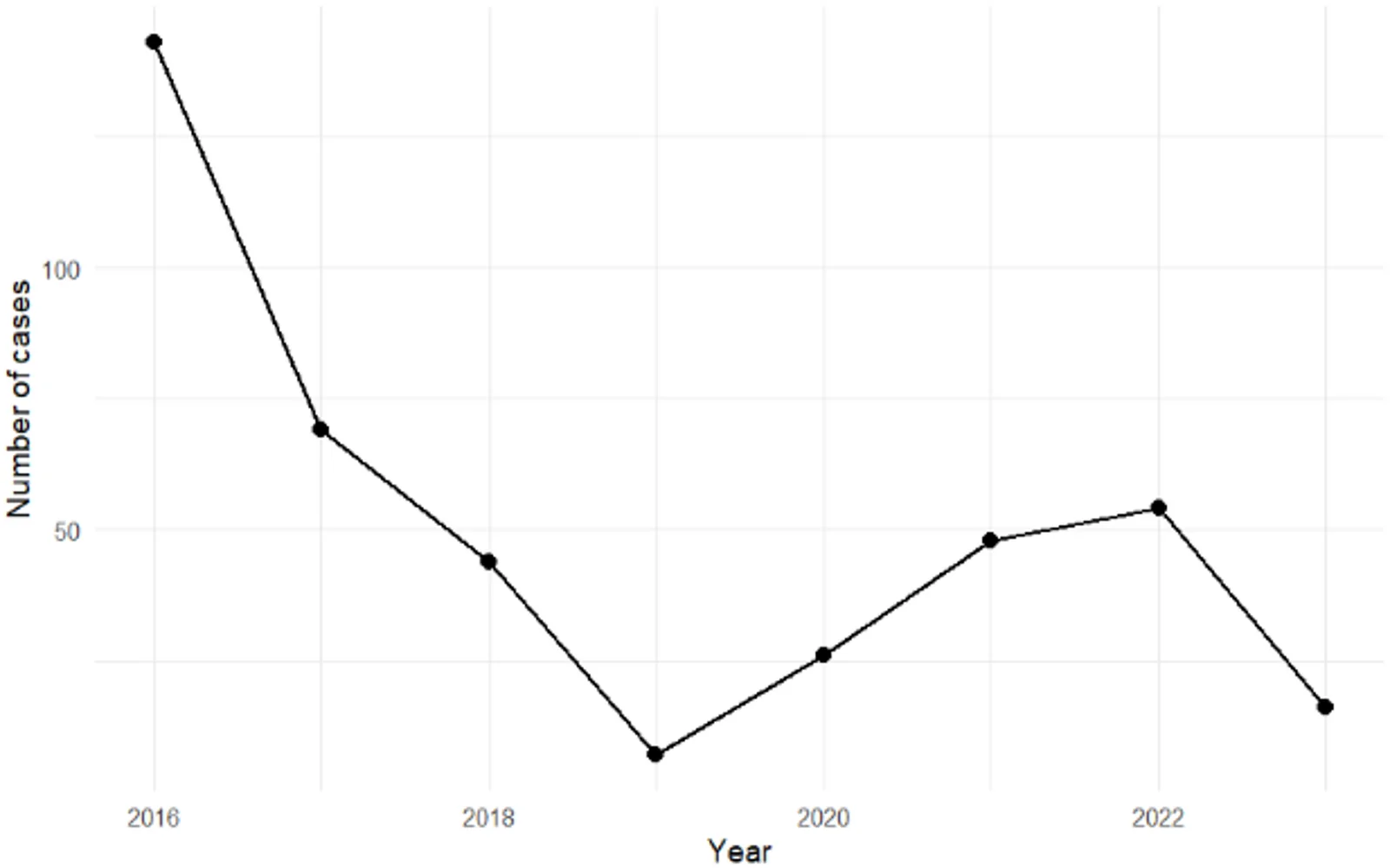
Annual suspected and confirmed CSM cases in the Upper West Region of Ghana, 2016–2023

Case counts varied substantially across years, with the highest number reported in 2016 (143), followed by a decline through 2019 [7]. Case numbers increased again from 2020 onward before declining in 2023 (Fig. 1). The high number of cases recorded in 2016 likely reflects the large meningitis outbreak documented in northern Ghana and the Upper West Region during that period. Spatially, most consultations originated from Wa Municipal (43.7%), Wa West (31.4%), and Wa East (14%) districts.

Comparison of included and excluded observations (Supplementary Table S4) showed that included observations were older on average than excluded observations (19.3 vs. 10.6 years, *p* < 0.001) and had lower symptom count (1.7 vs. 2.6, *p* < 0.001). There were no significant differences in sex, laboratory confirmation status, or days to consultation.

### Bivariate associations

In unadjusted analyses (Supplementary Table S1), mortality differed significantly by age group and clinical presentation. Older age groups and altered consciousness were associated with higher mortality, while classic meningitis symptoms such as fever, headache, and neck stiffness were not significantly associated with mortality in bivariate analyses. Mortality did not differ significantly by sex or laboratory confirmation status.

The exploratory symptom-count variable and selected clinical features were associated with delay to consultation in bivariate analyses. Moderate symptom count (2–3 symptoms) and neck stiffness showed significant associations with delay to consultation, while age group, sex, laboratory confirmation status, and most individual symptoms were not significantly associated.

### Factors associated with mortality

Table 2 presents results from the multivariable logistic regression model examining factors associated with mortality among patients with suspected or confirmed CSM.

**Table 2:** Factors associated with mortality among suspected and confirmed cerebrospinal meningitis cases in the Upper West Region of Ghana, 2016–2023 (*N* = 335)

Variables	aOR	95% CI	*p*-value
**Age group** (years)			
0–15 (*Reference*)			
16–45	4.73**	1.636–13.705	0.004
≥ 46	6.01**	1.608–22.46	0.008
**Days to Consultation**	1.01	0.909–1.122	0.853
**Sex**			
Female (*Reference*)			
Male	0.96	0.424–2.169	0.92
**Laboratory result**			
Suspected (*Reference*)			
Confirmed positive	2.07	0.931–4.618	0.074
**Clinical presentation**			
No symptoms recorded (*Reference*)			
Classical	0.38*	0.151–0.972	0.044
Neurological	3.16	0.780–12.791	0.107
Other non-specific	3.84*	1.332–11.05	0.013

aOR = adjusted odds ratio; CI = confidence interval. Reference categories are indicated. Estimates

are from multivariable logistic regression with robust standard errors. *p < 0.05; **p < 0.01; ***p < 0.001

Age was strongly associated with mortality. Compared with patients aged 0–15 years, those aged 16–45 years had significantly higher odds of death (adjusted odds ratio [aOR] = 4.7, 95% CI: 1.64–13.7), as did patients aged ≥ 46 years (aOR = 6.0, 95% CI: 1.61–22.46).

Clinical presentation was also associated with mortality. Using patients with no symptoms recorded as the reference group, those presenting with classic meningitis symptoms had lower odds of death (aOR = 0.4, 95% CI: 0.15–0.97). In contrast, patients presenting with other non-specific symptoms had significantly higher odds of death (aOR = 3.8, 95% CI: 1.33–11.05). Presentation with neurological manifestations was also associated with higher odds of death, although this association did not reach statistical significance (aOR = 3.2, 95% CI: 0.78–12.80).

Delay to consultation was not significantly associated with mortality. Sex and laboratory confirmation status were also not significantly associated with mortality.

Additional descriptive analysis among the 32 mortality cases showed that the median time from symptom onset to healthcare consultation was 2.0 days (IQR: 0–3.5 days; mean: 2.3 days, SD: 2.2). 25% of patients who died sought care on the same day of symptom onset, while 75% presented within 3.5 days (Supplementary Table S5).

Findings were consistent across sensitivity analyses with district fixed effects and Firth’s penalized logistic regression, with similar direction, magnitude, and statistical significance of key associations (Supplementary Table S2).

Additional analyses examining combinations of symptom presentations showed that patients presenting with only non-specific symptoms had significantly higher odds of mortality compared with those presenting with classic symptoms only (aOR: 8.0; 95% CI: 1.12–57.15). Similarly, patients presenting with both classic and non-specific symptoms also had increased odds of death (aOR: 4.6; 95% CI: 1.31–16.32) (Supplementary Table S6).

### Factors associated with delay to consultation

In secondary analyses examining delay to consultation (Table 3), the exploratory symptom-count variable was significantly associated with longer delays in seeking care. Compared with patients with no symptoms recorded, those with moderate symptom count (2–3 symptoms) experienced longer delays to consultation (β = 1.9 days, 95% CI: 0.70–3.08), as did those with high symptom count (4 or more symptoms) (β = 0.8 days, 95% CI: 0.31–1.31).

**Table 3:** Factors associated with delay to consultation among suspected and confirmed cerebrospinal meningitis cases in the Upper West Region of Ghana, 2016–2023 (*N* = 336)

Variables	β (days)	95% CI	*p*-value
**Age group** (years)			
0–15 (*Reference*)			
16–45	0.16	-0.535–0.849	0.656
≥ 46	-0.46	-1.182–0.263	0.212
**Sex**			
Female (*Reference*)			
Male	0.125	-0.535–0.785	0.71
**Laboratory result**			
Suspected (*Reference*)			
Confirmed positive	-0.317	-0.87–0.235	0.26
**Symptom count**			
No symptoms recorded (*Reference*)			
Low symptom count (1)	0.047	-0.884–1.979	0.92
Moderate symptom count (2–3)	1.888**	0.695–3.082	0.002
High symptom count (4+)	0.808**	0.311–1.305	0.002

β = regression coefficient (days); CI = confidence interval. Reference categories are indicated. Estimates are from a multivariable ordinary least squares (OLS) regression with robust standard errors. *p < 0.05; **p < 0.01; ***p < 0.001

Age, sex, and laboratory confirmation status were not significantly associated with delay to consultation.

Sensitivity analyses produced estimates consistent in both direction and magnitude with the primary model. Specifically, when relaxing parametric assumptions via OLS with bootstrapped standard errors, or employing an alternative generalized linear model, our conclusions remain unchanged (Supplementary Table S3).

## Discussion

Ghana is affected by CSM in a seasonal and predictable fashion; a systematic review found the pooled prevalence of meningitis in Ghana to be 16.22% during non-outbreak periods and 23.91% during outbreak periods [7]. UWR records CSM cases every year. A study conducted between 2015 and 2019 in the entire northern Ghana revealed that UWR consistently recorded the highest number of cases between 2017 and 2019 [9], with our study revealing a total of 407 surveillance-defined suspected and confirmed CSM cases over the 8-year study period.

The findings of our study show that there was a slight male preponderance in the incidence of CSM (55.8%) which is consistent with several previous studies in Ghana [7–9, 15] that showed 50.8–55.3% of incident cases to be male. In addition, a surveillance study of CSM in the UWR of Ghana between 2015 and 2021 showed 50.82% of all cases to be males and the remainder (49.18%) female. However, a Ghanaian study revealed a female majority in incident cases of CSM cases in 2016 with a male to female ratio of 1:1.3 [16], while another study conducted in the UWR from 2009 to 2013 [12] found that 52% of cases were females. Although the reasons for the slight male majority in cases remain unclear and were not analyzed in this study, possible explanations include differences in health-seeking behaviours, living conditions, and socialization patterns between the sexes in northern Ghana.

Although vaccination in Ghana and UWR in particular has resulted in an appreciable drop in incident cases [12], CSM still affects all ages, and this is reflected in our findings. This study found that about 9 in 10 cases recorded were aged 0–45 years old. Children and young adults have a higher prevalence of CSM [7, 12, 16, 17] and it has been suggested that this is because they are more likely to be in large gatherings and enclosed environments such as dormitories, parties, and other social events, increasing their likelihood of contracting the infection [7, 18].

The case fatality rate of CSM has dropped within the last 20 years, largely as a direct consequence of effective antibiotic therapy and vaccination [19], although a third of meningitis survivors develop neurodevelopmental sequelae [20], while 20% of all affected cases either die or survive with disabling complications [19]. This study found an overall case fatality rate of 7.9%. A study in Ghana reported an unusually low case fatality rate of 0.82% [16]. Although the reasons were not explicitly stated by the authors of that paper, a possible reason for such a low case fatality rate could be the dilution of confirmed or actual CSM cases with suspected or unconfirmed cases, possible prompt case management, and possibly low confirmation of actual deaths. The case fatality rate in the Northern region of Ghana is 9.7% [21], while that of neighboring Upper East Region is 6.1% [15]. A similar study conducted in the UWR showed a case fatality rate of 12.2%, but in individuals aged 70+, it was as high as 25% [12]. This is consistent with the findings in this study which showed that case fatality in older people was the highest among all the age groups at 15.9%, while children aged 0–15 years, though representing over half of all reported cases, had substantially lower mortality (3.1%) in keeping with the rate of 7% found in another study [22].

Fever, neck stiffness, headache and/or altered consciousness have for decades been referred to as the “classic” meningitis symptoms and they continue to be the most reported symptoms of meningitis [9, 23]. Interestingly, this classic presentation of symptoms occurs at the same time in just around half the time – 53.1% in our study and 44% in another study [24], however, 95% of patients in that study exhibited at least two symptoms of headache, neck stiffness, fever, and altered mental status. Another study involving 493 participants showed that around two thirds of adults had the classic presentation, but all of the study participants had at least one of these symptoms, with 95% having at least a fever and 88% having at least neck stiffness [25]. In this study it was found that majority of patients had fever (41.0%), neck stiffness (35.3%), and headache (31.7%), consistent with previous studies [9, 23, 25]. Altered consciousness and convulsions/seizures were reported in 5.5% and 14.3% of cases, respectively, according to our findings. Seizures occur in up to 23% of patients with meningitis [25], and this proportion is similar to what was found by our study. These findings emphasize the fact that CSM has specific clinical features that, although possessing a relatively low sensitivity, have a high specificity that should aid clinicians in maintaining a high index of suspicion in highly prevalent areas to drive investigation and appropriate treatment.

The finding that mortality was strongly associated with age was in agreement with previous literature [13, 24, 25–27] where it is seen that the older an individual is, the more likely they are to die if they contract CSM, supporting our current understanding of the dynamics of disease. An older person, relative to a younger one, generally has a weaker immune system; the likelihood of comorbidities that might negatively impact their fight against infections; and a higher risk of delayed diagnosis due to atypical presentations. Furthermore, although not statistically significant, presentation with neurological manifestations such as seizures and altered consciousness was associated with higher odds of death. This is in agreement with other studies that found that a low Glasgow Coma Score (GCS) and seizures carried a worse prognosis and were associated with mortality [25, 27, 28], likely because any individual presenting with neurological symptoms may be at an advanced stage of the disease.

Interestingly, this study found that other non-specific CSM symptoms (such as body weakness and pain, cough, and vomiting), were associated with increased mortality while the classic meningitis symptoms were associated with reduced mortality. This means that a person who presents with headache, neck stiffness, and fever was less likely to die. Indeed, a previous study found that patients who eventually died were less likely to complain of symptoms such as headache, nausea, or vomiting [27]. This may be because the classic symptoms in and of themselves do not relate to disease severity, and hence cannot determine prognosis. In addition, patients with either the classic or neurological symptoms are very likely to be assessed quicker by a physician since those symptoms are specific for CSM. Furthermore, the public is generally educated on the classic features of CSM, and an individual who develops neck stiffness and a fever is more likely to present for care than one who develops a cough and body pain.

Delay to consultation (and consequently evaluation and treatment), was not significantly associated with mortality, contrasting with a previous study [13] that found that treatment delay was associated with increase in 30-day mortality. A study conducted in Zambia found that reasons for delays to seeking care for CSM included electing to start treatment of symptoms at home, issues related to transportation to health facilities including distances and cost, and referral delays [29], while another study conducted in neighbouring Upper East Region revealed that participants later diagnosed with CSM only visited healthcare facilities when they were very sick, with 18% choosing to receive treatment at home [30]. Other contributors to delay in treatment included old age, presence of co-morbidities such as stroke, and suspicion of infections other than meningitis [13]. In this study, about half of the cases sought health care within a day of symptom onset, reflecting prompt presentation. As with many other life-threatening infections, delayed care for CSM might lead to poorer outcomes, although the extent of delay, together with illness severity and other modulating factors such as age, co-morbidity, and immune status might influence the outcome. Interestingly, patients with two or more symptoms recorded had longer delays to consultation. This may reflect the progressive nature of CSM, where additional symptoms may become documented as illness evolves. However, because the symptom-count variable was based on available surveillance symptom fields and did not capture symptom severity, duration, or timing, it should be interpreted as the breadth of recorded symptoms rather than clinical severity. The early symptoms of CSM are mostly non-specific, and so affected individuals might assume they have other ailments such as a cold or malaria, and would only present for care when they experience more symptoms. Furthermore, due to poverty and constrained healthcare access, many affected people may prefer to self-medicate or stay at home until the disease progresses to multiple symptoms, prompting them to seek care [31]. Age, sex, and laboratory confirmation status were not significantly associated with delay to consultation.

Although males are slightly more affected in several studies, sex was not associated with mortality in this study, consistent with findings from Denmark [13].

This study has some limitations. Firstly, reliance on surveillance data limited control over data quality and the scope of available variables. Information on comorbidities, vaccination history, socioeconomic factors (such as income and education), health system barriers, and treatment was not available, limiting our ability to examine their potential influence on outcomes. In addition, missing data resulted in the exclusion of some observations from multivariable analyses, and differences in age and symptom count between included and excluded observations suggest the possibility of selection bias that should be considered when interpreting the findings. Several regression estimates exhibited wide confidence intervals, likely reflecting the relatively small number of deaths and sparse observations in specific clinical-presentation subgroups. While this limited statistical efficiency reduces the precision of the estimated results, thus resulting in wider margins of uncertainty around the coefficients, it does not alter the expected value or direction of the reported magnitudes, which remain unbiased. Nonetheless, these wider intervals should be considered when interpreting the magnitude of the reported associations.

Secondly, not all cases had laboratory confirmation. Although clinically suspected cases were identified based on established surveillance definitions, incomplete laboratory confirmation introduces diagnostic uncertainty, as some cases may have represented meningitis from other etiologies, or other clinical conditions with similar presentations. However, laboratory confirmation status was accounted for in the analyses, and sensitivity analyses restricted to laboratory-confirmed cases produced findings consistent in direction and magnitude with the primary model, supporting the robustness of the results.

Finally, measurement limitations related to surveillance data should be considered. Symptoms were abstracted from available symptom fields in the routine surveillance dataset and did not capture symptom severity, timing, duration, progression, or all presenting complaints. Therefore, the category of no symptoms recorded should not be interpreted as confirmed absence of symptoms or clinical complaints. In addition, Ghana Health Service surveillance records may arise through routine clinical presentation, referral, active outbreak surveillance, or contact-tracing activities, but the dataset did not include a variable identifying the route through which each record entered surveillance. We therefore could not distinguish actively identified or contact-traced records from routine clinical presentations. These limitations may influence the interpretation of the symptom-count and clinical-presentation variables. In addition, the dataset reflects cases managed within the Upper West regional healthcare system and includes referrals from two districts in the Savannah Region, while no cases from Sissala East and Sissala West districts were recorded in the available data over the study period.

Despite these limitations, this analysis provides valuable insights into the patterns of CSM cases in a high-prevalence area and the factors that are associated with mortality. Future research should study post-discharge outcomes in Ghana and qualitatively examine individuals’ understanding of CSM and its symptoms, and the factors that contribute to delayed presentation for care.

## Conclusions

CSM is highly prevalent in the northern part of Ghana. It can be infectious and rapidly fatal without appropriate intervention. The disease affects all ages: although the young are disproportionately affected, older individuals and those with symptoms other than the classic meningitis symptoms have higher odds of mortality.

It is important that outbreak response strategies move beyond routine case counts to the incorporation of assessment of mortality risks among different demographics. Policymakers have to be cognizant of the fact that CSM remains an important public health concern, and that vaccinations; health education and promotion especially before and during outbreaks; and targeted interventions for older populations should be prioritized in the bid to fight CSM.

The public should be educated that CSM can present with non-specific symptoms, and individuals should seek prompt care during outbreaks, as such presentations are associated with higher mortality. Clinicians in the high-prevalence areas should consider the fact that high-risk individuals might present with atypical symptoms during outbreak periods and since this group may carry a higher mortality, a higher index of suspicion should be maintained.

## Supplementary Information

Below is the link to the electronic supplementary material.

Supplementary Material 1

## Data Availability

The datasets used and/or analyzed during this study are publicly available and can be obtained with permission from Ghana’s Integrated Disease Surveillance and Response (IDSR) system and the District Health Information Management System (DHIMS).

## Abbreviations

CSM: Cerebrospinal meningitis
GCS: Glasgow coma scale
UWR: Upper west region
IDSR: Integrated disease surveillance and response
