# Supplementary material for: Epidemiology, clinical outcomes, and factors associated with mortality among cerebrospinal meningitis cases in the Upper West Region of Ghana: an eight-year surveillance study
Source: BMC Infect Dis. 2026 Jul 22;26:1823. doi: 10.1186/s12879-026-14070-3 (PMC13679026; doi:10.1186/s12879-026-14070-3)
Supplement: Supplementary file 1 [file 12879_2026_14070_MOESM1_ESM.docx]

*Supplementary Table S1. Bivariate associations between clinical characteristics and (A) mortality and (B) delay to consultation among suspected and confirmed cerebrospinal meningitis cases in the Upper West Region of Ghana, 2016–2023*

A. **Mortality**

| **Variable** | **Survived (n)** | **Died (n)** | **Mortality (%)** | **Chi-Square (df)** | **p-value** |
| --- | --- | --- | --- | --- | --- |
| **Symptom count** |  |  |  | 2.897 | 0.408 |
| No symptoms recorded | 110 | 11 | 9.09% |  |  |
| Low symptom count (1) | 8 | 2 | 20.00% |  |  |
| Moderate symptom count (2-3) | 66 | 6 | 8.33% |  |  |
| High symptom count (4+) | 190 | 13 | 6.40% |  |  |
| **Age Group** |  |  |  | 16.173 | **0.000***** |
| 0-15 years | 218 | 7 | 3.11% |  |  |
| 16-45 Years | 119 | 18 | 13.14% |  |  |
| 46+ years | 37 | 7 | 15.91% |  |  |
| **Altered Consciousness** | |  |  | 15.584 | **0.000***** |
| No | 321 | 21 | 6.14% |  |  |
| Yes | 14 | 6 | 30.00% |  |  |
| **Convulsion** |  |  |  | 2.696 | 0.101 |
| No | 284 | 26 | 8.39% |  |  |
| Yes | 51 | 1 | 1.92% |  |  |
| **Neck Stiffness** |  |  |  | 0.049 | 0.825 |
| No | 218 | 17 | 7.23% |  |  |
| Yes | 117 | 10 | 7.87% |  |  |
| **Headache** |  |  |  | 0.062 | 0.804 |
| No | 228 | 19 | 7.69% |  |  |
| Yes | 107 | 8 | 6.96% |  |  |
| **Fever** |  |  |  | 0.002 | 0.963 |
| No | 197 | 16 | 7.51% |  |  |
| Yes | 138 | 11 | 7.38% |  |  |
| **Final Test Results** |  |  |  | 2.237 | 0.135 |
| Suspected | 198 | 16 | 7.48% |  |  |
| Confirmed | 114 | 16 | 12.31% |  |  |
| **Sex** |  |  |  | 0.005 | 0.945 |
| Female | 166 | 14 | 7.78% |  |  |
| Male | 208 | 18 | 7.96% |  |  |

B. **Delay to consultation**

| **Variable** | **Comparison (reference vs group)** | **χ² / Fisher’s Exact** | **p-value** |
| --- | --- | --- | --- |
| Sex | Male (1) vs Female (0) | 2.717 | 0.437 |
| Laboratory result | Confirmed (1) vs Suspected (0) | 4.375 | 0.224 |
| **Age group (overall)** |  | 4.727 | 0.579 |
| 0–15 | Yes (1) vs No (0) | 1.592 | 0.661 |
| 16–45 | Yes (1) vs No (0) | 2.358 | 0.501 |
| ≥46 | Yes (1) vs No (0) | 2.76 | 0.43 |
| **Symptom count (overall)** | 4 categories | 2.897 | 0.408 |
| No symptoms recorded | Yes (1) vs No (0) | 12.523 | 0.006** |
| Low symptom count (1) | Yes (1) vs No (0) | 3.678 | 0.298 |
| Moderate symptom count (2–3) | Yes (1) vs No (0) | 8.23 | 0.041* |
| High symptom count (4+) | Yes (1) vs No (0) | 2.733 | 0.435 |
| **Individual Symptoms** |  |  |  |
| Fever | Yes (1) vs No (0) | 6.537 | 0.088 |
| Headache | Yes (1) vs No (0) | 2.056 | 0.561 |
| Neck stiffness | Yes (1) vs No (0) | 11.323 | 0.010** |
| Altered consciousness | Yes (1) vs No (0) | 3.585 | 0.31 |

Chi-square or Fisher’s exact tests were used to assess bivariate associations between categorical variables. Fisher’s exact test was applied when expected cell counts were small. For mortality analyses, comparisons are between patients who died and those who survived. For delay to consultation, comparisons reflect differences across categories. Symptom count reflects the number of symptoms recorded in the surveillance dataset and should not be interpreted as a clinical severity score. Symptoms represent those recorded at the time of first consultation and do not capture symptom progression following presentation. Reference categories are indicated.

*Supplementary Table S2. Sensitivity analyses for factors associated with mortality among suspected and confirmed cerebrospinal meningitis cases in the Upper West Region of Ghana, 2016–2023*

|  | Logit FE Robust SE | Firthlogit |
| --- | --- | --- |
| VARIABLES | aOR (95% CI), p-value | aOR (95% CI), p-value |
| **Age group** (years) |  |  |
| 0-15 (*Reference*) |  |  |
| 16-45 | 4.931** | 4.296** |
|  | (1.549 - 15.698) p=0.007 | (1.689 - 10.924) p=0.002 |
|  |  |  |
| ≥46 | 7.011** | 5.523** |
|  | (1.817 - 27.053) p=0.005 | (1.755 - 17.383) p=0.003 |
|  |  |  |
| **Days to Consultation** | 0.967 | 1.036 |
|  | (0.794 - 1.178) p=0.740 | (0.942 - 1.140) p=0.468 |
|  |  |  |
| **Sex** |  |  |
| Female (*Reference*) |  |  |
| Male | 1.158 | 0.946 |
|  | (0.470 - 2.854) p=0.749 | (0.441 - 2.029) p=0.887 |
| **Laboratory result** |  |  |
| Suspected (*Reference*) | |  |
| Confirmed Positive | 2.126 | 1.2 |
|  | (0.917 - 4.933) p=0.079 | (0.934 - 4.267) p=0.074 |
|  |  |  |
| **Clinical presentation** | |  |
| No symptoms recorded (*Reference*) | |  |
| Classical | 0.411 | 0.413 |
|  | (0.137 - 1.234) p=0.113 | (0.155 - 1.102) p=0.077 |
|  |  |  |
| Neurological | 3.31 | 3.035 |
|  | (0.665 - 16.483) p=0.144 | (0.966 - 9.532) p=0.057 |
|  |  |  |
| Other non-specific | 4.801* | 3.517* |
|  | (1.251 - 18.425) p=0.022 | (1.307 - 9.461) p=0.013 |
|  |  |  |

aOR = adjusted odds ratio; CI = confidence interval. Reference categories are indicated.
Estimates are from multivariable logistic regression models including district fixed effects with robust standard errors and Firth’s penalized likelihood logistic regression to assess robustness to small-sample bias and potential separation.
**p* < 0.05; **p < 0.01; ****p* < 0.001

*Supplementary Table S3. Sensitivity analyses for factors associated with delay to consultation among suspected and confirmed cerebrospinal meningitis cases in the Upper West Region of Ghana, 2016–2023*

|  | OLS Bootstrapp | GLM |  |
| --- | --- | --- | --- |
| VARIABLES | β (95% CI), p-value | β (95% CI), p-value |  |
| **Age group** (years) |  |  |  |
| 0-15 (*Reference*) |  |  |  |
| 16-45 | 0.157 | 0.032 |  |
|  | (-0.545 - 0.859) p=0.622 | (-0.233 - 0.298) p=0.811 |  |
|  |  |  |  |
| ≥46 | -0.460 | -0.160 |  |
|  | (-1.204 - 0.285) p=0.266 | (-0.563 - 0.242) p=0.435 |  |
| **Sex** |  |  |  |
| Female (*Reference*) |  |  |  |
| Male | 0.125 | 0.126 |  |
|  | (-0.531 - 0.781) p=0.709 | (-0.127 - 0.380) p=0.329 |  |
|  |  |  |  |
| **Laboratory result** |  |  |  |
| Suspected (*Reference*) |  |  |  |
| Confirmed positive | -0.317 | -0.114 |  |
|  | (-0.857 - 0.222) p=0.249 | (-0.376 - 0.148) p=0.393 |  |
|  |  |  |  |
| **Symptom count** |  |  |  |
| No symptoms recorded (*Reference*) | |  |  |
| Low symptom count (1) | 0.047 | 0.099 |  |
|  | (-0.947 - 1.041) p=0.926 | (-0.635 - 0.833) p=0.792 |  |
|  |  |  |  |
| Moderate symptom count (2–3) | 1.888** | 0.822*** |  |
|  | (0.702 - 3.075) p=0.002 | (0.469 - 1.174) p<0.001 |  |
|  |  |  |  |
| High symptom count (4+) | 0.808** | 0.452** |  |
|  | (0.334 - 1.282) p=0.001 | (0.164 - 0.741) p=0.002 |  |

β = regression coefficient (days); CI = confidence interval. Reference categories are indicated. Estimates are from ordinary least squares (OLS) regression with bootstrap standard errors and generalized linear models (GLM). *p < 0.05; **p < 0.01; ***p < 0.001.

*Supplementary Table S4. Comparison of included and excluded observations used in multivariable analyses.*

| Variable | | Excluded observations (Group Mean) | Included observations (Group Mean) | p-value |
| --- | --- | --- | --- | --- |
| Age (years) | 10.6 | | 19.3 | <0.001 |
| Symptom count | 2.55 | | 1.74 | <0.001 |
| Male sex (%) | 46.50% | | 57.70% | 0.083 |
| Laboratory-confirmed CSM (%) | 55.60% | | 37.20% | 0.264 |
| Days to consultation | 3.06 | | 2.29 | 0.163 |

*Supplementary Table S5. Descriptive statistics for delay to consultation among mortality cases (n=32).*

| Mortality outcome | Mean | SD | Median | 25th percentile | 75th percentile |
| --- | --- | --- | --- | --- | --- |
| Survived | 2.4 | 3.8 | 2 | 1 | 3 |
| Died | 2.3 | 2.2 | 2 | 0 | 3.5 |
| Overall | 2.4 | 3.7 | 2 | 1 | 3 |

*Supplementary Table S6. Association between symptom presentation profiles and mortality among suspected and confirmed cerebrospinal meningitis cases in the Upper West Region of Ghana, 2016–2023*

| Symptom Profile | Deaths/Total (%) | Adjusted Odds Ratio | 95% CI | p-value |
| --- | --- | --- | --- | --- |
| Classic symptoms only | 4/125 (3.2%) | 1.00 (Reference) | — | — |
| Non-specific symptoms only | 2/15 (13.3%) | 7.99* | 1.12‒57.15 | 0.038 |
| Both (Classic and Non-Specific | 10/90 (11.1%) | 4.62* | 1.31‒16.32 | 0.017 |
| Neither classic nor non-specific symptoms recorded | 16/176 (9.1%) | 2.37 | 0.75‒7.46 | 0.142 |

*aOR = adjusted odds ratio; CI = confidence interval. Estimates are from multivariable logistic regression adjusted for age group, sex, laboratory confirmation status, and days to consultation.*
